# Detection and Inheritance Pattern of Copy Number Variations (CNVs) in Children with Multiple Congenital Anomalies

**DOI:** 10.1186/1755-8166-7-S1-I35

**Published:** 2014-01-21

**Authors:** Frenny Sheth

**Affiliations:** 1 FRIGE’s Institute of Human Genetics, FRIGE House, Satellite, Ahmedabad, India

## 

Microscopically visible chromosomal alterations or segmental aneusomy are one of the major causes for congenital anomalies and require cytogenetic investigations. Conventional G-banding analysis is limited to the detection of imbalances greater than 5-10 Mb. Expressivity of a phenotype generally correlates with the degree of genetic imbalance, therefore, patients with a greater genetic imbalance are more likely to be investigated, which creates a diagnostic bias. This does not reflect the full range of phenotypic presentation that may be associated with an imbalance of a particular chromosomal segment. Systematic clinical diagnosis of a rare syndrome can be carried out using FISH to characterize known deletion/duplication breakpoints or by screening for known micro-deletion syndromes and sub-telomeric imbalances where routine banding technique suggests “normal” karyotype. In the past decade, array- Comparative Genomic Hybridization (a-CGH) has made it feasible to detect cryptic imbalances in more number of cases with apparently normal chromosomal makeup in addition to characterize structurally rearranged chromosome. The study of inheritance pattern by a combination of conventional cytogenetic and a-CGH techniques has helped to determine the imbalance as either *de novo* or inherited. a-CGH could also provide information on whether an imbalance is pathogenic or polymorphic, which could aid in calculating the recurrent risk for future pregnancies. It provides several advantages over conventional cytogenetic techniques such as whole genome coverage in a single experiment with resolution of 20-150 kb, faster turnaround time, higher sensitivity and specificity and non-requirement of dividing cells. Having an extensive coverage of the genome and high resolution, this technique has been pinned as the “first line genetic test” to study cryptic genetic imbalances in cases with developmental delay and congenital anomalies, where G-banded karyotype is “normal”.

